# Thickness-stiffness trade-off improves lodging resistance in rice

**DOI:** 10.1038/s41598-023-37992-3

**Published:** 2023-07-04

**Authors:** Satoru Tsugawa, Hiroyuki Shima, Yukitaka Ishimoto, Kazuya Ishikawa

**Affiliations:** 1 Department of Mechanical Engineering, Faculty of Systems Science and Technology, Akita Prefectural University, Yurihonjo, Akita 015-0055 Japan; 2 Department of Environmental Sciences, University of Yamanashi, Kofu, Yamanashi 400-8510 Japan; 3 Department of Genomics and Breeding, Iwate Biotechnology Research Center, Kitakami, Iwate 024-0003 Japan; 4 Graduate School of Science and Technology, Nara Institute of Science and Technology, Ikoma, Nara 630-0192 Japan; 5 Ritsumeikan Global Innovation Research Organization, Ritsumeikan University, Kusatsu, Shiga 525-8577 Japan

**Keywords:** Plant breeding, Computational biophysics

## Abstract

Lodging of cereal crops significantly reduces grain yield and quality, making lodging resistance a prime target for breeding programs. However, lodging resistance among different rice (*Oryza sativa* L.) cultivars in the field remains largely unknown, as is the relationship between the major properties of culms such as their morphological and mechanical properties. Here, we investigated the morphological and mechanical properties of 12 rice cultivars by considering different internodes within culms. We detected variation in these two traits among cultivars: one set of cultivars had thicker but softer culms (thickness-type), while the other set of cultivars showed stiffer but thinner culms (stiffness-type). We designate this variation as a thickness-stiffness trade-off. We then constructed a mechanical model to dissect the mechanical and/or morphological constraints of rice culms subjected to their own weight (self-weight load). Through modeling, we discovered that ear weight and the morphology of the highest internode were important for reducing deflection, which may be important factors to achieve higher lodging resistance. The mechanical theory devised in this study could be used to predict the deflection of rice culms and may open new avenues for novel mechanics-based breeding techniques.

## Introduction

Rice (*Oryza sativa* L.) is an important staple food in many countries, especially in Asia and Africa. Achieving high productivity in rice is a major goal in agriculture^1,2^, but high-yielding rice cultivars often face mechanical difficulties such as lodging of the aboveground stems called culms. Previous studies have identified three types of lodging: (1) culm bending, where the culm bends too much and cannot bear the load of leaves and grains; (2) culm breaking, where the culm breaks due to an excessive applied load; and (3) root lodging, where uprooting occurs when root anchorage is not sufficient^2,3^. Rice culms can bend or break due to their morphological properties, such as thin and/or long internodes^4–9^, the length from the ground to the ears^8^, the dry weight per unit length^10^, and thin leaf sheaths^11^, as well as from environmental pressures, such as wind and rainfall^12^. Therefore, it is fundamental to understand the mechanisms underlying the different lodging behaviors and deploy this knowledge to identify or engineer a cultivar with both high yield and high lodging resistance.

Several methods have been proposed to evaluate the mechanical resistance of different types of lodgings. The lodging rate (percentage of lodging in a paddy field) often has been used for evaluating all lodging types, although it does not offer precise control over test conditions and thus measures mechanical resistance under varying weather conditions^13^. The pushing resistance is another method used to evaluate culm bending and breaking by measuring how the culm and the root resist to a pushing stimulation at the lowest internode of the culm^14,15^. The lodging index (LI), bending moment (weight × length), is used to evaluate lodging resistance in the context of culm breaking^10,16^. However, LI does not distinguish between lodging resistance to bending and that to breaking^17^. Recently, the bending moment of the internode at breaking (BMB) was proposed as a novel quantitative parameter^2,18^. Nevertheless, it is still unclear which parameter is more suitable for evaluating the lodging resistance from culm bending and/or breaking.

In the field of botany, plants have often assumed to be an elastic material^19,20^. The bending of standing culms can be formulated as a post-buckling behavior of elastic columns with a large deformation under a self-weight condition. Different types of mechanical theories have been reported in trees^21^ and in other heavily elastic columns^19,20,22–27^ to describe post-buckling. Few of these theories have proposed methodologies evaluating the lodging resistance by quantifying the relative contributions of self-weight and ear weight against bending rigidity^23,25–27^. Since these are non-dimensional parameters, a comparison of different cultivars with different culm heights would be extremely informative to relate these mechanical studies to the lodging resistance of rice culms and to build a theoretical model inspired by actual data.

For data analysis, we used the knowledge accumulated in the breeding research field. In previous studies, two genes associated with lodging resistance were detected in one *indica* and one tropical *japonica* rice variety^28,29^. In addition, the chromosomal regions associated with lodging resistance have been identified using a backcrossed inbred line population derived from a cross between the temperate *japonica* Nipponbare and *aus* Kasalath cultivars that was scored for pushing resistance^14^. Cultivars of temperate *japonica* also share chromosomal regions involved in the regulation of culm thickness^30^. Thus, genetic analysis using different rice cultivars is a promising method to accelerate breeding for lodging resistance. However, it remains unknown which morphological and mechanical properties of culms are important to lodging resistance.

In this article, we first summarize the morphological and mechanical properties of rice culms from 12 different cultivars, which revealed two types of culms in these cultivars: thickness-type culms and stiffness-type culms. We then constructed a mechanical theory and implemented the resulting model using real morphological data measured above to evaluate the degree of deflection, clarifying important parameters for lodging resistance. Finally, we discuss the morphological and mechanical constraints of rice culms and explore how to increase the lodging resistance of rice culms.

## Methods

### List of rice cultivars

The list is summarized in Supplementary Table S1. We have permission to collect the rice cultivars (*Oryza sativa* L.) from Genebank of the National Agricultural Research Organization (NARO) in Japan.

### Plant materials and cultivation

The *japonica* rice (*Oryza sativa*) cultivar Hitomebore and other breeding varieties collected from the National Agriculture and Food Research Organization World Rice Core Collection^36^ (Supplementary Table S1) were grown in the paddy rice field of Iwate Biotechnology Research Center during the summer of 2020. Approximately 30 days after heading, eight main culms were used for phenotypic measurements. All methods were carried out in accordance with relevant guidelines.

### Morphological measurements

The outer and inner diameters of internodes were measured by the following method. We first cut off the middle of each internode, stamped their cross-sectional surface onto paper, and measured it using a ruler.

For the mechanical test of rice culms and the definitions of maximum point loading and maximum point displacement, Young’s modulus, maximum point loading (MPL), and maximum point displacement (MPD) were measured by a three-point bending test using a force tester (MCT-2150; A&D, Tokyo, Japan). Each internode was placed on two fulcrums with a 4-cm spatial interval, and the load was applied to the center of the samples. MPL and MPD were measured when the internode broke. Young’s modulus was calculated by detecting the slope between stress and strain. Stress and strain were calculated by the following equations:

$${\text{Stress}} = {\text{load}} \times {\text{8L}}_{{\text{v}}} {\text{D}}_{{\text{o}}} /\pi \left( {{\text{D}}_{{\text{o}}}^{4} - {\text{D}}_{{\text{i}}}^{4} } \right)$$

$${\text{Strain}} = {\text{displacement}} \times {\text{6D}}_{{\text{o}}} /{\text{L}}_{{\text{v}}}^{2}$$

where L_v_ (= 4 cm) is the distance between fulcrums, D_o_ is the outer diameter, and D_i_ is the inner diameter.

### Derivation of derivatives to calculate the deflection of the culm

We used a Maclaurin series expansion of the inclination angle for the case of $\frac{d{\theta }_{i}({s}_{i}=0)}{d{s}_{i}}=0$ and $\frac{d{\theta }_{i}({s}_{i}=0)}{d{s}_{i}}=c \left(\mathrm{const}\right)$ for the internode i as described in the supplementary information.

## Results

### Thickness-stiffness trade-off is found in the properties of rice culms

**Figure 1:**
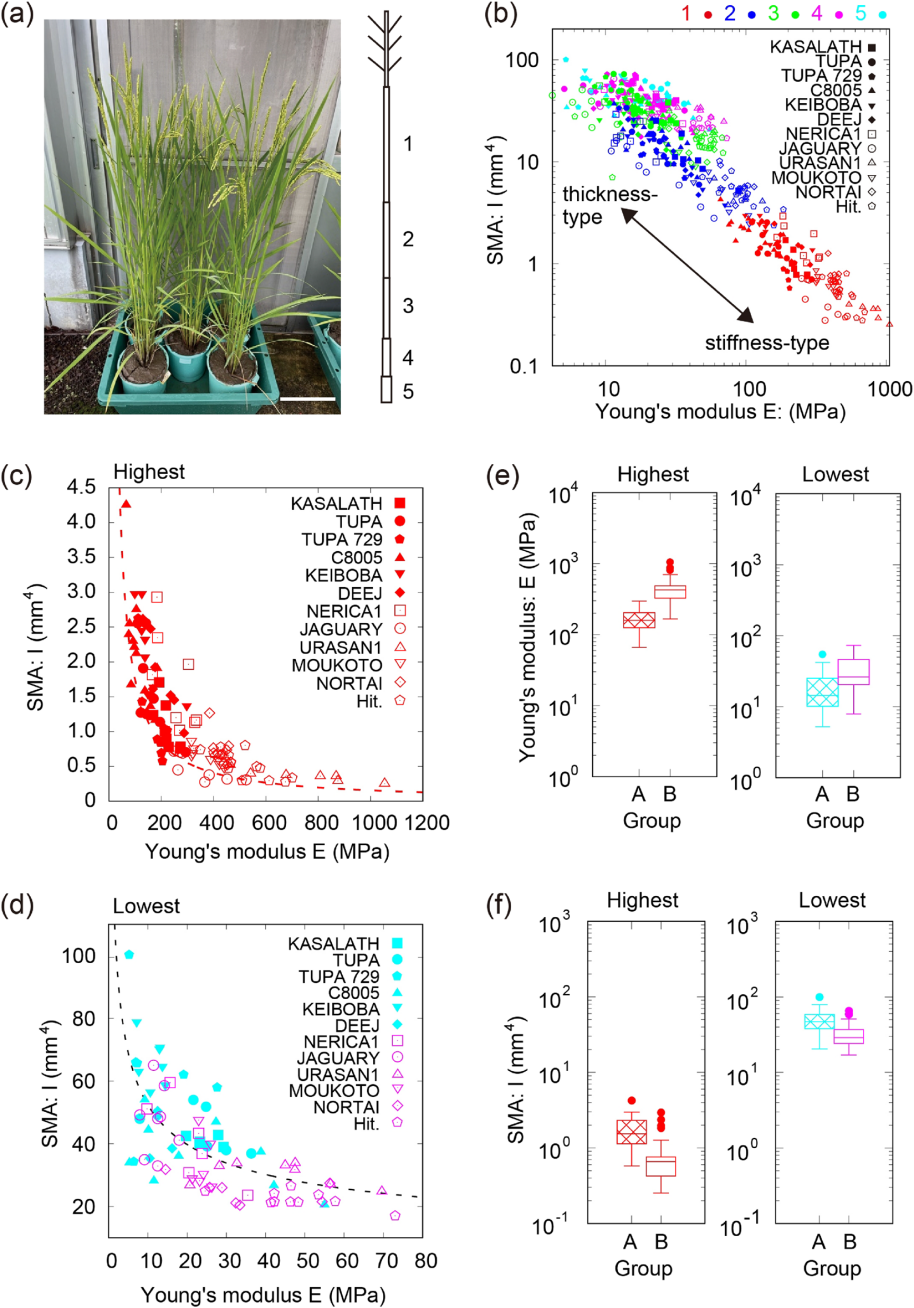
Morphological and mechanical characteristics of rice culms. (**a**) Photograph of rice culms from the cultivar Hitomebore (left) and schematic illustration of internodes of rice culms (right). Scale bar = 10 cm. (**b**) Log–log plot of second moment of area (SMA) as a function of Young’s modulus for all internodes. The colors show the order of internode from first (red), second (blue), third (green), fourth (magenta), and fifth internode (cyan). Group A is represented by filled symbols, and group B is represented by open symbols. (**c**, **d**) SMA as a function of Young’s modulus for the highest (**c**) or lowest internode (**d**). The fitting functions are shown as dotted lines with the functions $I\sim {E}^{{A}_{1}}$ (${A}_{1}$= –1.05, standard error [SE] = 0.08) at the highest internode and $I\sim {E}^{{A}_{2}}$ (${A}_{2}$= –0.268, SE = 0.08) at the lowest internode. (**e**) Boxplot of Young’s modulus of the highest and lowest internodes. (**f**) Boxplot of SMA of the highest and lowest internodes.

To quantify the morphological and mechanical parameters of the rice culms (Fig. 1a), we measured the lengths and outer/inner radii of internodes and then calculated the second moment of area (SMA denoted by variable I). We also determined Young’s modulus (denoted by variable E), maximum point loading (MPL), and maximum point displacement (MPD) with three-point bending tests (see Methods, Fig. 1b and all the data in Figs. S1–S12). We measured these properties across different cultivars from group A, which have relatively taller culms up to five internodes below the ears (KASALATH, TUPA121-3 [TUPA], TUPA729, C8005, KEIBOBA, and DEEJAOHUALUO [DEEJ]), and from group B, which have relatively shorter culms up to four internodes below the ears (New Rice for Africa1 [NERICA1], JAGUARY, URASAN1, MOUKOTO, NORTAI, and Hitomebore). For brevity, we denote the number of the lowest internodes as b (5th in group A and 4th in group B) in this study.

The bending rigidity D ($=\mathrm{E}\times \mathrm{I}$) was larger in the lower internodes than in the higher internodes, indicating that the lower internodes have a higher lodging resistance than the higher internodes (Supplementary Fig. S13). We also investigated the contributions of E and I to D by drawing the corresponding scatterplot between these parameters for all cultivars (Fig. 1b). The 12 rice cultivars loosely clustered at two diverged types of culms: thickness-type with thicker and softer internodes and stiffness-type with thinner and stiffer internodes. We call this divergence a thickness–stiffness trade-off consisting of D. When qualitatively assessed by manually pinching the internode, the lower internodes can be classified as thickness-type rather than the higher internodes; lower internodes are therefore thicker but softer. Interestingly, at the highest and lowest internodes, almost all cultivars from group A tended to belong to the thickness-type, while those from group B were largely from the stiffness-type (Fig. 1c–f).

### Resistance to bending lodging of the lowest internode does not depend on the height of the culms

As each culm is of different size, we considered size-independent (non-dimensional) information in terms of shape and mechanics to compare the different cultivars.

As a non-dimensional parameter for shape, we used the slenderness ratio for internode i defined as

1 $${\lambda }_{i}=\frac{{L}_{i}}{{R}_{i}}, \left(i=1,...,b\right),$$

where ${L}_{i}$ is the length of each internode i, and ${R}_{i}={I}_{i}/{A}_{i}$ is the radius of gyration of area with the cross-sectional area ${A}_{i}$. The SMA of each internode ${I}_{i}$ is defined as ${I}_{i}=({\left({r}_{i}^{\left(out\right)}\right)}^{4}-{\left({r}_{i}^{\left(in\right)}\right)}^{4})/4$. Associated with Euler buckling criterion with fixed basal end^31^, the critical top load on internode i was formulated as

2 $${F}_{cr}=\frac{{\uppi }^{2}{E}_{i}{I}_{i}}{4{L}_{i}^{2}}=\frac{{\pi }^{2}{E}_{i}{A}_{i}^{2}}{4{\lambda }_{i}^{2}{I}_{i}}.$$

Therefore, a smaller slenderness ratio is beneficial because of the relatively shorter associated length; thus, index ${\lambda }_{i}$ represents a degree of lodging with a morphological origin where a small value means a high resistance against lodging. Therefore, we considered ${\lambda }_{i}$ the shape safety index.

As a non-dimensional parameter for mechanics, we used the elasto-gravity relativity associated with the relative contributions of tip-weight and self-weight to bending rigidity^23,25–27^ defined as

3 $${\mathrm{\alpha }}_{\mathrm{i}}=\frac{{F}_{i}{L}_{i}^{2}}{{E}_{i}{I}_{i}}, {\upbeta }_{\mathrm{i}}=\frac{{\rho }_{i}{L}_{i}^{3}}{{E}_{i}{I}_{i}}, \left(i=1,\ldots ,b\right),$$

where the concentrated load for internode *i* is ${F}_{1}=W$, ${F}_{i}=W+{\sum }_{j=1}^{i-1}{\rho }_{j}{L}_{j} (i=2,...,b)$, W is ear weight, and ${\rho }_{i}$ is the self-weight per unit length of internode i. The meanings of α and β are the relative ratios of concentrated load at the tip of the internode and the self-weight divided by the bending stiffness, respectively, which allow for a systematic comparison of lodging resistance between internodes in the same individual or between different internodes from different individuals. The important mechanical parameter for the internode i should be the total elasto-gravity relativity ${\alpha }_{i}+{\beta }_{i}$ because it represents the relativity to the bending rigidity of all the weight of internode i. By definition, a smaller total elasto-gravity relativity is beneficial because of a relatively lighter weight or relatively higher bending rigidity. Thus, the index ${\alpha }_{i}+{\beta }_{i}$ represents the degree of lodging with a mechanical origin where a small value means a high resistance against lodging. Therefore, we named it the mechanical safety index.

**Figure 2:**
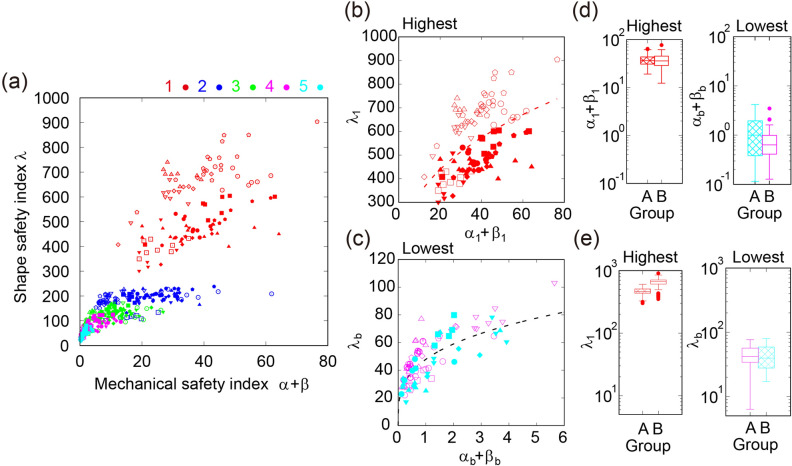
Non-dimensional indices for the comparison of lodging across different rice cultivars. (**a**) Shape safety index as a function of mechanical safety index for all internodes. (**b**, **c**) Shape safety index as a function of mechanical safety index for the highest internode (**b**) or for the lowest internode (**c**). The fitting functions are shown with ${\uplambda }_{1}\sim {\left({\alpha }_{1}+{\beta }_{1}\right)}^{{B}_{1}}$(${B}_{1}$= 0.387, standard error [SE] = 0.06) and ${\uplambda }_{\mathrm{b}}\sim {\left({\alpha }_{b}+{\beta }_{b}\right)}^{{B}_{2}}$(${B}_{2}$= 0.387, SE = 0.03). Pearson’s correlation coefficients were 0.51 (highest) and 0.68 (lowest). (**d**, **e**) Boxplot of the mechanical safety index and the shape safety index of the highest internode (**d**) and the lowest internode (**e**).

Based on the actual data of rice culms, we determined that the shape safety index and the mechanical safety index vary among different cultivars. It is important to note that lower internodes have a smaller shape safety index than higher internodes, suggesting that the culms reinforce their body, especially at the lower internodes (Fig. 2a), which is consistent with the literature^4–9^. At the highest internode position, the mechanical safety index in group A was comparable to that in group B, but the shape safety index in group A was smaller than that in group B, reflecting different shape properties of the highest internode between groups A and B (Fig. 2b, d). In addition, we also noticed that both properties at the lowest internodes are comparable in groups A and B (Fig. 2c, e), suggesting that the properties of the lowest internode do not depend on the height of the culms.

### Breaking lodging resistance of the lowest internode does not depend on the height of the culms

We next tested whether resistance to breaking lodging depended on the height of culms. To this end, we calculated the modulus of rupture (MOR)^32,33^. The MOR of internode i was defined as

4 $${\mathrm{MOR}}_{i}=\mathrm{MPL}\cdot \frac{{L}_{i}{r}_{i}^{\left(out\right)}}{4{I}_{i}}.$$

**Figure 3:**
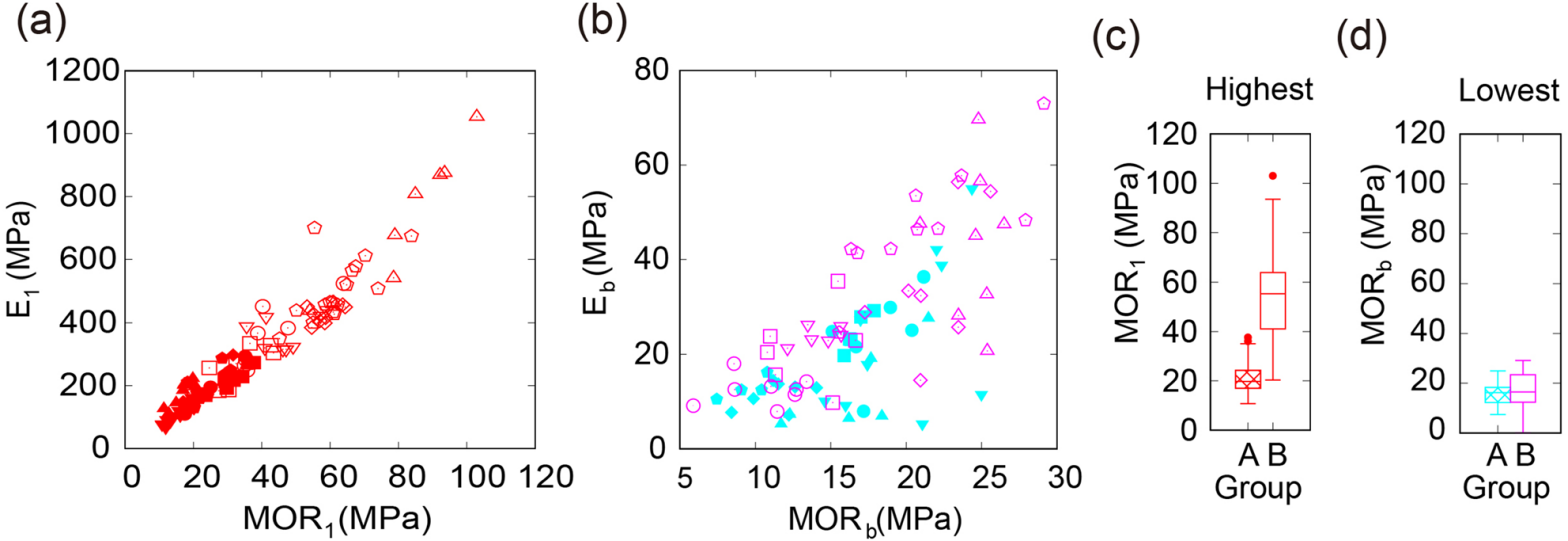
Relationship between bending property and breaking property of rice culms. (**a**, **b**) MOR (modulus of rupture) as a function of Young’s modulus for the highest (**a**) or lowest internode (**b**). (**c**, **d**) Boxplot of the MOR of the highest internode (**c**) or the lowest internode (**d**).

By definition, a large MOR is beneficial because of the relatively higher weight needed to reach the breaking point; this index thus represents the degree of breaking with a breaking origin, where a large value means a strong breaking resistance. To compare the different cultivars, we plotted ${\mathrm{MOR}}_{i}$ as a function of the Young’s modulus. As expected, the MOR was positively correlated with Young’s modulus for highest and lowest internodes (Fig. 3a, b, respectively), indicating that the breaking property is almost directly and linearly proportional to the bending property for all cultivars tested. Interestingly, at the highest internode, the MOR was lower in cultivars from group A than from group B, but was similarly low at the lowest internode for both groups (Fig. 3c, d). These data suggest that the breaking property of the lowest internode does not depend on the height of the culms.

### A mechanical model of an elastic column with many internodes ensures a quantitative assessment of the mechanical safety index

**Figure 4:**
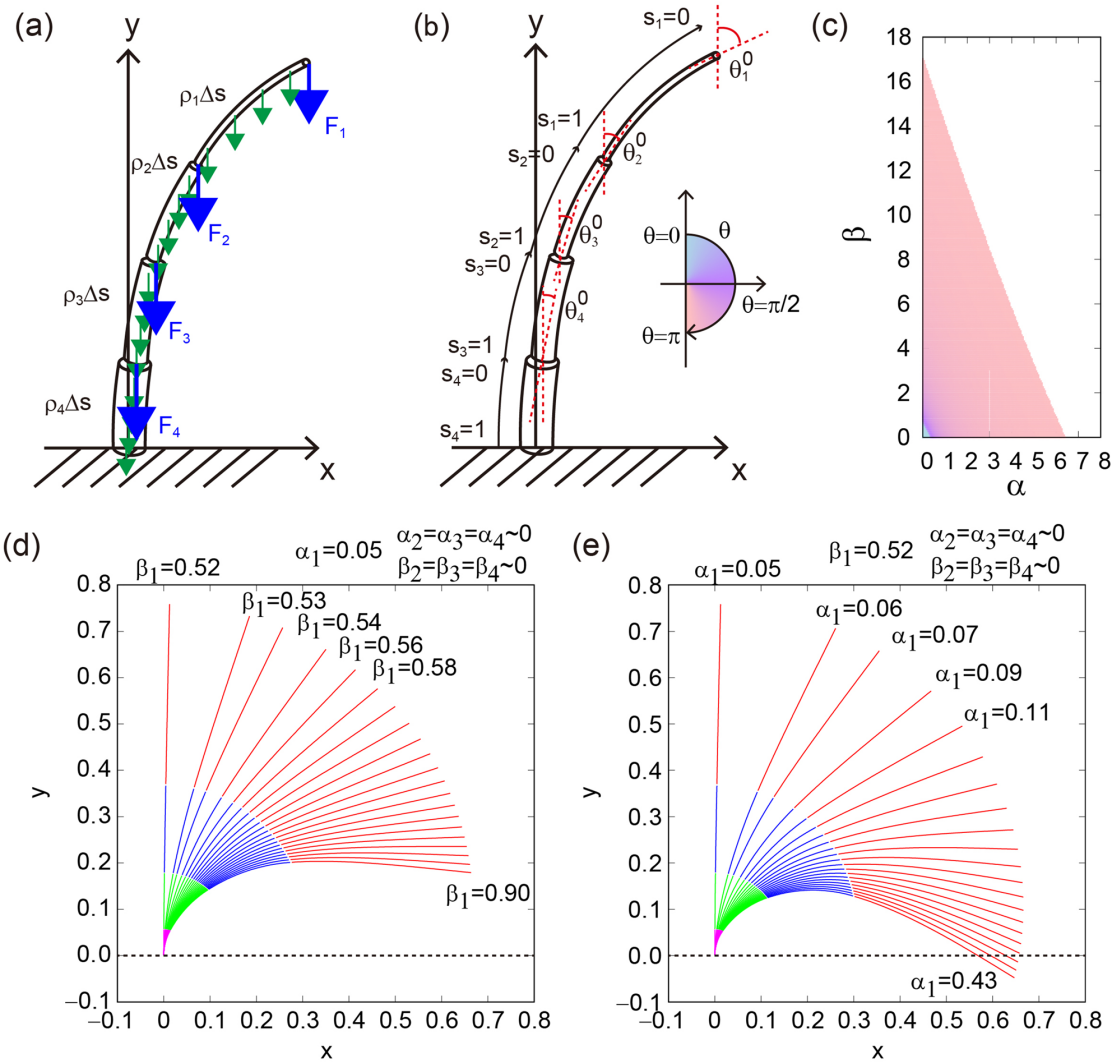
Inference of deflected morphology of rice culms derived from the elastic column theory compared to actual data. (**a**) Force components on each internode. (**b**) Definition of angle for each internode. (**c**) Inference of deflected morphology by the elastic column theory as a function of α and β. (**d**, **e**) Resulting shapes of the theoretical model as a function of the index ${\alpha }_{1}$ for internode ${\alpha }_{1}=0.05,{\alpha }_{2}={\alpha }_{3}={\alpha }_{4}\sim 0,{\beta }_{2}={\beta }_{3}={\beta }_{4}\sim 0$ (**d**) and the index ${\beta }_{1}$ for internode 1 with ${\beta }_{1}=0.52,{\alpha }_{2}={\alpha }_{3}={\alpha }_{4}\sim 0,{\beta }_{2}={\beta }_{3}={\beta }_{4}\sim 0$ (**e**).

We formulated a mechanical model based on a vertically built heavy elastic column according to a previous study^27^. We considered an elastic material with up to four or five internodes (a schematic illustration with four internodes is shown in Fig. 4a, b). The morphological and mechanical properties (e.g., length, radius, and Young’s modulus) of internodes can be different rather than a fixed value, which is different from the previous study^27^. The i-th internode is subject to both a concentrated load at the tip of internode ${F}_{i}$ and a uniformly distributed weight (${\rho }_{i}\Delta s$) (Fig. 4a). The local bending moment ${M}_{i}$ obeys the following equation,

5 $$\frac{{\mathrm{dM}}_{\mathrm{i}}\left({l}_{i}\right)}{d{l}_{i}}=\left\{{F}_{i}+{\rho }_{i}\left({L}_{i}-{l}_{i}\right)\right\}\mathrm{sin}{\theta }_{i}\left({l}_{i}\right), \left(i=\mathrm{1,2},\cdots ,b\right),$$

where the variable ${l}_{i}$ is the arc length along the curve measured from the bottom of the internode i, and ${s}_{i}(=1-{l}_{i}/{L}_{i})$ is the normalized variable (Fig. 4b). The index ${\theta }_{i}({s}_{i})$ is an inclination angle at position ${s}_{i}$ ranging from ${\theta }_{i}=0$ in the vertical upward direction to ${\theta }_{i}=\pi /2$ in the horizontal one. The local curvature can be formulated as

6 $${E}_{i}{I}_{i}\frac{d{\theta }_{i}\left({l}_{i}\right)}{d{l}_{i}}=-{M}_{i}\left({l}_{i}\right), \left(i=\mathrm{1,2},\cdots ,b\right).$$

Using the variables and non-dimensional parameters ${\alpha }_{i}$ and ${\beta }_{i}$ discussed above, the governing equations were written as,

7 $$\frac{{d}^{2}{\theta }_{i}\left({s}_{i}\right)}{d{s}_{i}^{2}}=-\left({\alpha }_{i}+{\beta }_{i}{s}_{i}\right)\mathrm{sin}{\theta }_{i}\left({s}_{i}\right), \left(i=\mathrm{1,2},\cdots ,b\right).$$

The boundary conditions were established as follows^27,34,35^:

8 $$\theta_{b} \left( {s_{b} = 1} \right) = 0\,{\text{at}}\,{\text{the}}\,{\text{lowest}}\,{\text{internode}}\,{\text{b}},$$

9 $$\theta_{i - 1} \left( {s_{i - 1} = 1} \right) = \theta_{i} \left( {s_{i} = 0} \right){\text{at}}\,{\text{the}}\,{\text{node}}\,{\text{between}}\,{\text{internodes}}\,{(}1 < i < b{),}$$

10 $${\text{E}}_{{{\text{i}} - 1}} I_{i - 1} \frac{{d\theta_{i - 1} \left( {s_{i - 1} = 1} \right)}}{{ds_{i - 1} }} = {\text{E}}_{{\text{i}}} I_{i} \frac{{d\theta_{i} \left( {s_{i} = 0} \right)}}{{ds_{i} }}{\text{at}}\,{\text{the}}\,{\text{node}}\,{\text{between}}\,{\text{internodes }}(1 < i < b),{\text{and}}$$

11 $$\frac{{d\theta_{1} \left( {s_{1} = 0} \right)}}{{ds_{1} }} = 0\,{\text{at}}\,{\text{the}}\,{\text{highest}}\,{\text{internode}}\,{1}.$$

The Maclaurin series representation of the solution can be constructed according to Reference^27^, written as,

12 $${\theta }_{i}\left({s}_{i}\right)={\theta }_{i}^{0}+{s}_{i}\frac{d{\theta }_{i}^{0}}{d{s}_{i}}+\frac{{s}_{i}^{2}}{2!}\frac{{d}^{2}{\theta }_{i}^{0}}{d{s}_{i}^{2}}+\frac{{s}_{i}^{3}}{3!}\frac{{d}^{3}{\theta }_{i}^{0}}{d{s}_{i}^{3}}+\cdots +\frac{{s}_{i}^{n}}{n!}\frac{{d}^{n}{\theta }_{i}^{0}}{d{s}_{i}^{n}}.$$

The series can be truncated at the ninth order for numerical accuracy^27^. The derivatives up to the ninth order are summarized in Methods. We solved Eq. (7) with the boundary conditions (8–11) numerically using the bisection method.

As a result, a color diagram of deflection angle (Fig. 4b) can be realized as functions of α and β (Fig. 4c). Culm morphology becomes more deflected with larger α and with larger β. With this theoretical evaluation, the rationality of indices α and β were quantitatively ensured by the elastic column theory. As discussed in the next section, we found that the parameter set (${\alpha }_{1}$ and ${\beta }_{1}$) for the highest internode is most important, we confirmed the parameter dependence to the standing shape of the culms in Fig. 4d, e, which demonstrate how the rice culms deflect depending on the parameter set.

### The length of the highest internode is most important for lodging resistance

From the above formulation, we showed that all the mechanical safety index ${\alpha }_{i}+{\beta }_{i}$ can affect the entire morphology of the culm. As the indices ${\alpha }_{i}$ and ${\beta }_{i}$ are comparable non-dimensional parameters, the higher magnitude indicates higher contribution to the deflection. Therefore, we examined which parameter most affected the safety index ${\alpha }_{1}+{\beta }_{1}$. The mechanical safety index is explicitly written as

13 $${\mathrm{\alpha }}_{1}+{\beta }_{1}=\frac{\left({F}_{1}+\rho {L}_{1}\right){L}_{1}^{2}}{{E}_{1}{I}_{1}}=\frac{4{L}_{1}^{2}\left\{W+\pi {\rho }^{^{\prime}}\left(\left({r}_{1}^{\left(out\right)2}-{r}_{1}^{\left(in\right)2}\right){L}_{1}\right)\right\}}{\pi {E}_{1}\left({\left({r}_{1}^{\left(out\right)}\right)}^{4}-{\left({r}_{1}^{\left(in\right)}\right)}^{4}\right)},$$

where $\mathrm{\rho{^{\prime}}}$ is the self-weight per unit volume.

The above safety index includes the parameters $W$, ${L}_{1}$, ${r}_{1}^{(out)}$, ${r}_{1}^{(in)}$, $\mathrm{\rho {^{\prime}}}$, and ${E}_{1}$; thus, these parameters should be focused. We then selected the Hitomebore cultivar as a representative culm to be considered. The average value of the ear weight W was ~ 3.5 × 10^−2^ (N), and the average value of the latter component of the numerator in Eq. (13) was $\rho {^{\prime}}{L}_{1}$=6.0 × 10^−3^ (N). Since ear weight has a larger order of magnitude, the ear weight W is dominant for lodging; thus, the total mechanical index is approximately reduced to the expression ${\mathrm{\alpha }}_{1}+{\beta }_{1}\simeq \frac{W{L}_{1}^{2}}{\pi {E}_{1}\left({\left({r}_{1}^{\left(out\right)}\right)}^{4}-{\left({r}_{1}^{\left(in\right)}\right)}^{4}\right)}.$

The sensitivity parameter can be estimated from the following variational equation:

14 $$\updelta \left({\mathrm{\alpha }}_{1}+{\upbeta }_{1}\right)=\frac{{L}_{1}^{2}}{\pi {E}_{1}\left({\left({r}_{1}^{\left(out\right)}\right)}^{4}-{\left({r}_{1}^{\left(in\right)}\right)}^{4}\right)}\delta W+\frac{2W{L}_{1}}{\pi {E}_{1}\left({\left({r}_{1}^{\left(out\right)}\right)}^{4}-{\left({r}_{1}^{\left(in\right)}\right)}^{4}\right)}\delta {L}_{1}-\frac{W{L}_{1}^{2}}{\pi {E}_{1}^{2}\left({\left({r}_{1}^{\left(out\right)}\right)}^{4}-{\left({r}_{1}^{\left(in\right)}\right)}^{4}\right)}\delta {E}_{1}-\frac{W{L}_{1}^{2}{\left({r}_{1}^{\left(out\right)}\right)}^{3}}{\pi {E}_{1}{\left({\left({r}_{1}^{\left(out\right)}\right)}^{4}-{\left({r}_{1}^{\left(in\right)}\right)}^{4}\right)}^{2}}\delta {r}_{1}^{\left(out\right)}+\frac{W{L}_{1}^{2}{\left({r}_{1}^{\left(in\right)}\right)}^{3}}{\pi {E}_{1}{\left({\left({r}_{1}^{\left(out\right)}\right)}^{4}-{\left({r}_{1}^{\left(in\right)}\right)}^{4}\right)}^{2}}\delta {r}_{1}^{\left(in\right)}$$

From the average values $W\sim 3.5\times {10}^{-2}$ N, ${L}_{1}\sim 3.7\times {10}^{-1}$ m, ${E}_{1}\sim 5.0\times {10}^{9}$N/m^2^, ${r}_{1}^{(out)}\sim 1.8\times {10}^{-3}$ m, and ${r}_{1}^{(in)}\sim 8.0\times {10}^{-4}$ m, we estimated 1% of their averages for the sensitivity analysis. We then evaluated the parameter sensitivity for each term (Table 1).

**Table 1:** Sensitivity of parameters.

W term	${L}_{1}$ term	${E}_{1}$ term	${r}_{1}^{(out)}$ term	${r}_{1}^{(in)}$ term
2.8 × 10^−5^	5.6 × 10^−4^	− 2.8 × 10^−4^	− 3.0 × 10^−4^	1.1 × 10^−5^

A positive value means a stronger contribution to the lodging resistance when it increases, and a negative value means a stronger contribution when it decreases.

We concluded that the most important parameter is ${L}_{1}$, with ${E}_{1}$ and ${r}_{1}^{(out)}$ as secondary important parameters, while the parameters $W$ and ${r}_{1}^{(in)}$ exhibited minor effects.

## Discussion

In this study, we analyzed the morphology and mechanics of rice culms and constructed a mechanical model to explain the mechanical rationality of the parameters α and β. We obtained the following results. (1) Internodes change from a stiffness-type to a thickness-type from the higher internode to the lower internode positions. (2) Rice cultivars from group A with taller culms and five internodes exhibited thickness-type internodes, while cultivars from group B with shorter culms and four internodes shared stiffness-type internodes. (3) Neither bending lodging resistance nor breaking lodging resistance appeared to depend on culm height. (4) Parameter sensitivity analysis showed that ear weight and the length of the first internode were important for lodging resistance.

We conceptualize the meaning of the trade-off between stiffness and thickness of the rice culm as follows. In general, plants extend their bodies from the soil upward, possibly with constant thickness and the same stiffness for each internode. However, our observations indicate that the lower internodes were thicker with a lower Young’s modulus, while the upper internodes were thinner with a higher Young’s modulus. As lower internodes become thicker, they may therefore modify their cell wall composition to maintain a balance between E and I. In addition, since biomass is limited and should be used efficiently for optimizing plant production under natural growth conditions, the thickness-stiffness strategy may emerge because there is an upper limit to increase bending rigidity more than necessary.

To our knowledge, it has been difficult to compare the lodging resistance among several rice cultivars due to the different heights of culms under different environmental conditions with the previously reported methods in the references^13–18^. In this study, to compare different internodes of different length, we successfully introduced ${\lambda }_{i}$ as a non-dimensional quantity representing shape and ${\alpha }_{i}+{\beta }_{i}$ as a non-dimensional quantity representing mechanics. Using these parameters, we were able to assess the lodging resistance of culms in terms of their shape and mechanics independently of their heights. Moreover, the mechanical model make it possible to infer the amount of deflection only from the set of parameters $\lambda$, α, and β. It should therefore also be possible to estimate approximate lodging resistance by simply examining $\lambda$, α, and β from actual data without solving mechanical equations, which is one of the highlights of this study. As this method is applicable to the other species, it may contribute to the understanding of lodging resistance of wheat and maize as well.

A practical use of the mechanical model is a prediction of the deflection angle of rice culms. For a typical condition where the parameters ${\alpha }_{1}$ and ${\beta }_{1}$ are dominant (Fig. 4c), the culms will lodge when ${\alpha }_{1}>0.16$ with ${\beta }_{1}=0.52$ or ${\beta }_{1}>0.84$ with ${\alpha }_{1}=0.05$ where the deflection angle ${\theta }_{0}$ reaches $\pi /2$ from the vertical position. The threshold of ${\theta }_{0}=\pi /2$ is determined by the sum $3{\alpha }_{1}+{\beta }_{1}\simeq 1$ which is consistent with the case of the single elastic column^27^. This threshold is extremely important because the danger of the lodging is determined by the threshold, i.e., when the ear weight increases (${\alpha }_{1}$ becomes large), a possible way to prevent lodging is to decrease the parameter ${\beta }_{1}$ by keeping the threshold. In practice, one can measure the quantities ${F}_{1}$, ${\rho }_{1}$, ${L}_{1}$, ${E}_{1}$, and ${I}_{1}$ to calculate the concrete values ${\alpha }_{1}$ and ${\beta }_{1}$ determining whether the culm will lodge when $3{\alpha }_{1}+{\beta }_{1}>1$ or will not lodge when $3{\alpha }_{1}+{\beta }_{1}<1$.

The mechanical model indicated that ear weight was important for lodging resistance (Eq. 13), suggesting that the length, thickness, and stiffness of the highest internode were also important because these parameters are directly influenced by ear weight. According to the analysis, the most sensitive parameter was the length of the first internode. In addition, ${r}_{1}^{(out)}$ was also an important parameter affecting sensitivity. These results demonstrated that the morphology of the first internode is most important for lodging resistance against their self-weight. This is consistent with the previously reported results, showing the importance of the stiffness of upper internodes (first to third) in rice has been reported due to the additional pressure imposed by the weight of neighboring rice^37–39^. Therefore, the identification and recombination of the genes and chromosomal regions, which are involved in the morphology of the first internode, may improve rice productivity in the future.

## Supplementary Information

Supplementary Information.

## Data Availability

The datasets used and/or analyzed during the current study are available from the corresponding author on reasonable request.
