# Supplementary material for: Thickness-stiffness trade-off improves lodging resistance in rice
Source: Sci Rep. 2023 Jul 4;13:10828. doi: 10.1038/s41598-023-37992-3 (PMC10319739; doi:10.1038/s41598-023-37992-3)
Supplement: Supplementary file 1 — Supplementary Information. [file 41598_2023_37992_MOESM1_ESM.pdf]

Supplementary Fig. S1. Internode length for all the data. L_e denotes the length of ear part, and L_i denotes the length of the internode i.

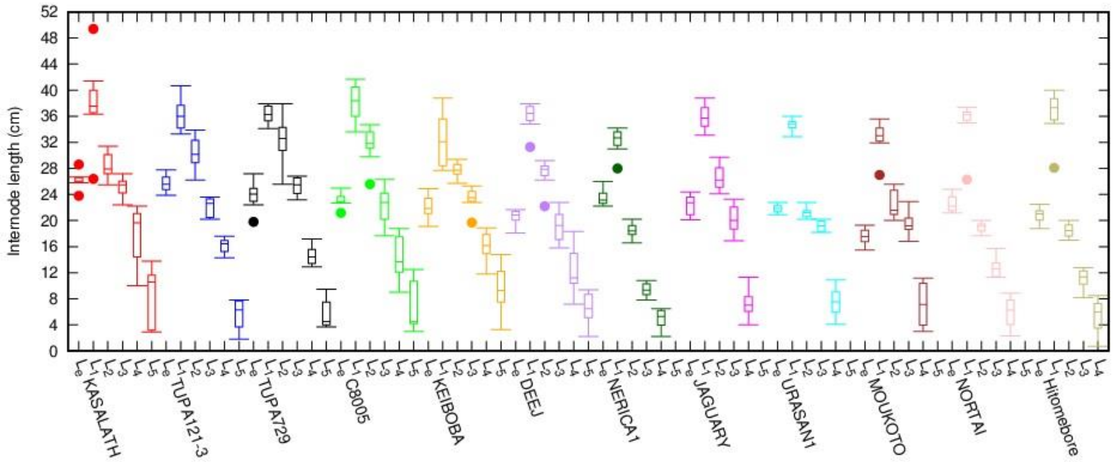

Supplementary Fig. S2. Inner diameter for all the data. di_i denotes the inner diameter of the internode i.

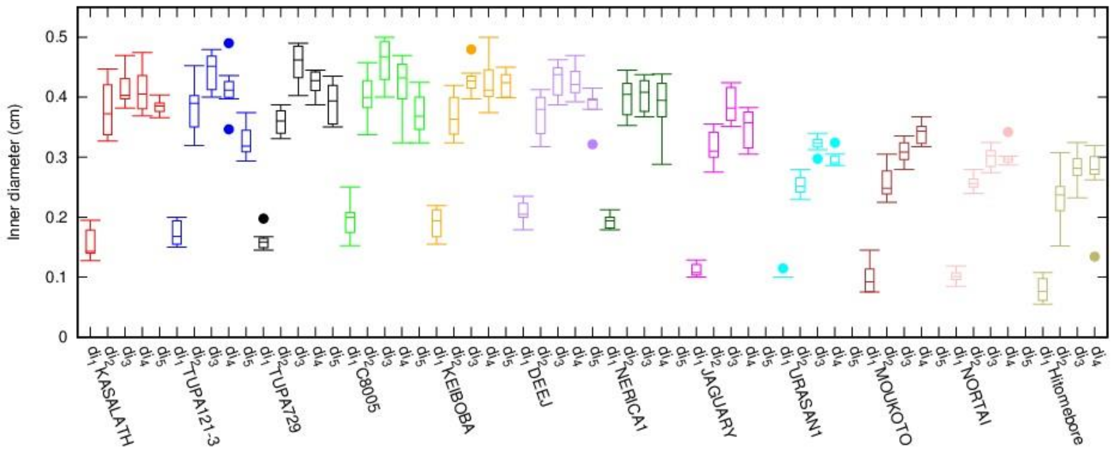

Supplementary Fig. S3. Outer diameter for all the data. do_i denotes the outer diameter of the internode i.

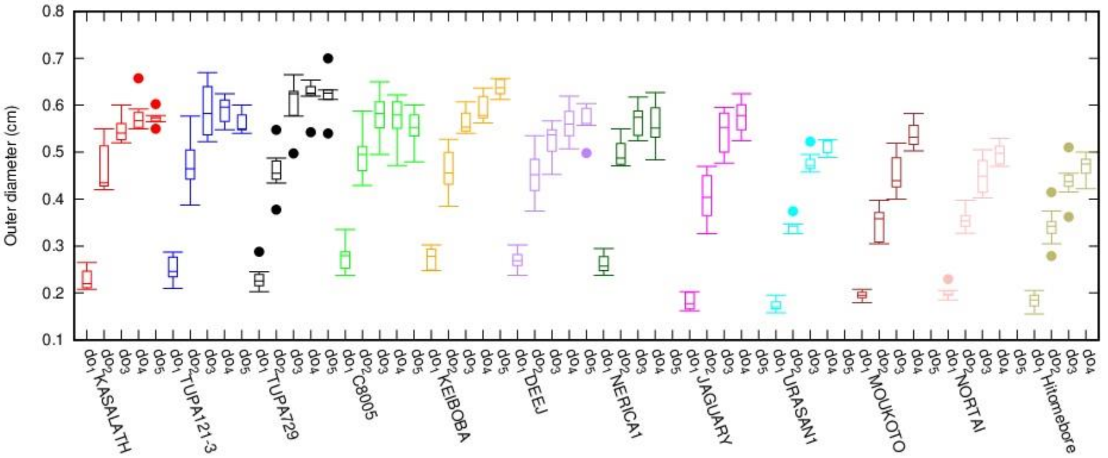

Supplementary Fig. S4. Internode thickness for all the data. T_i denotes the thickness of the internode i.

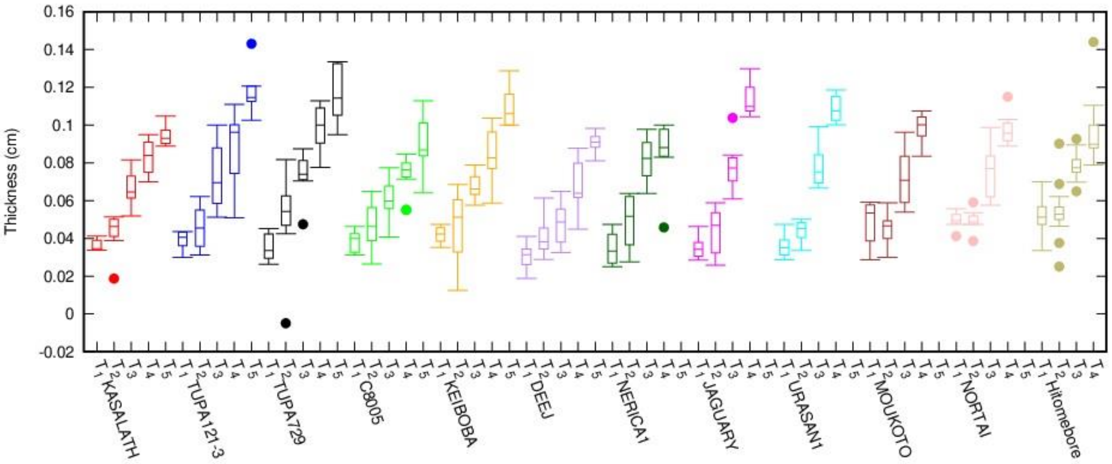

Supplementary Fig. S5. Cross-sectional area for all the data. A_i denotes the cross-sectional area of the internode i.

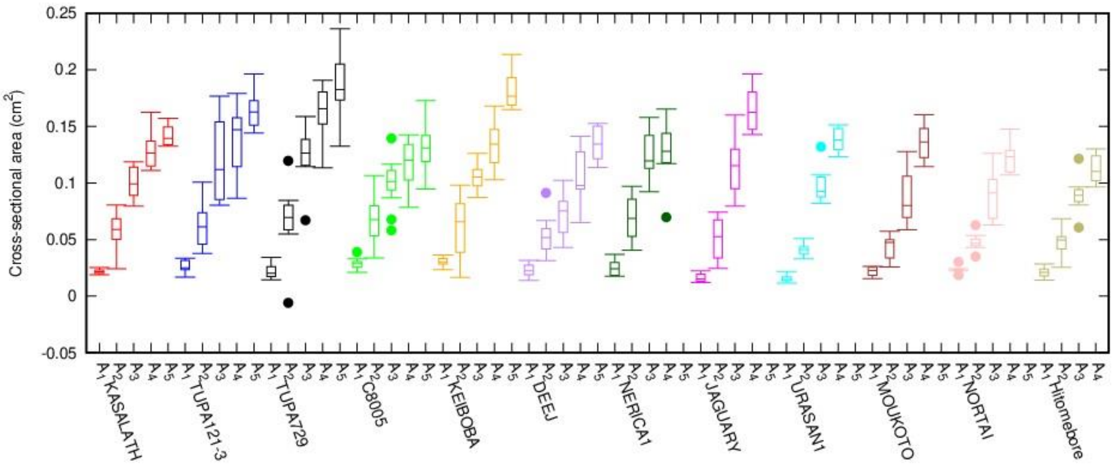

Supplementary Fig. S6. Section modulus for all the data. Z_i denotes the section modulus of the internode i.

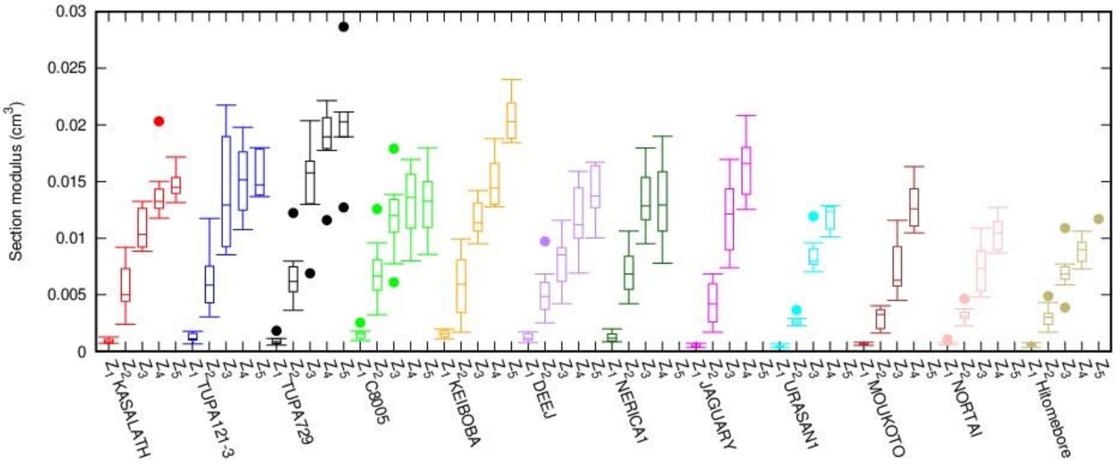

Supplementary Fig. S7. Second moment of area for all the data. I_i denotes the second moment of area of the internode i.

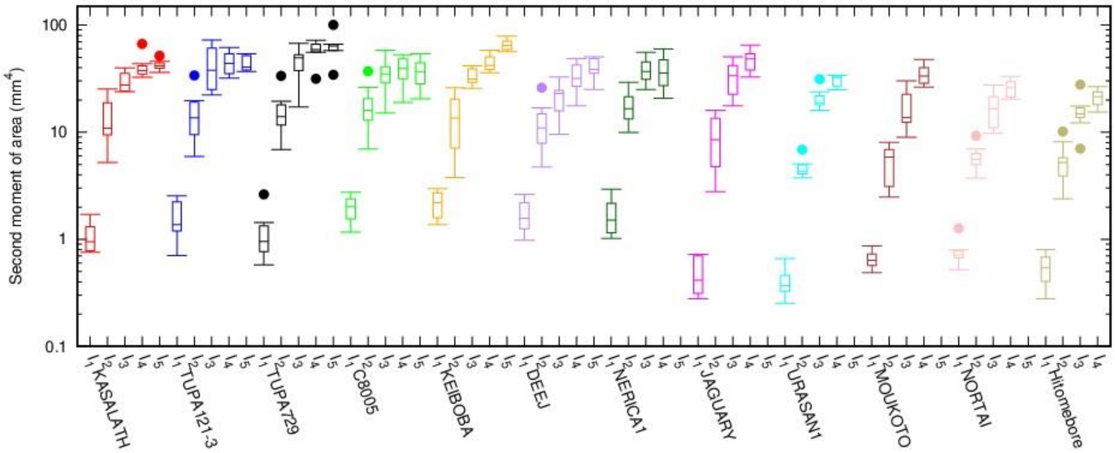

Supplementary Fig. S8. Young's modulus for all the data. E_i denotes Young's modulus of the internode i.

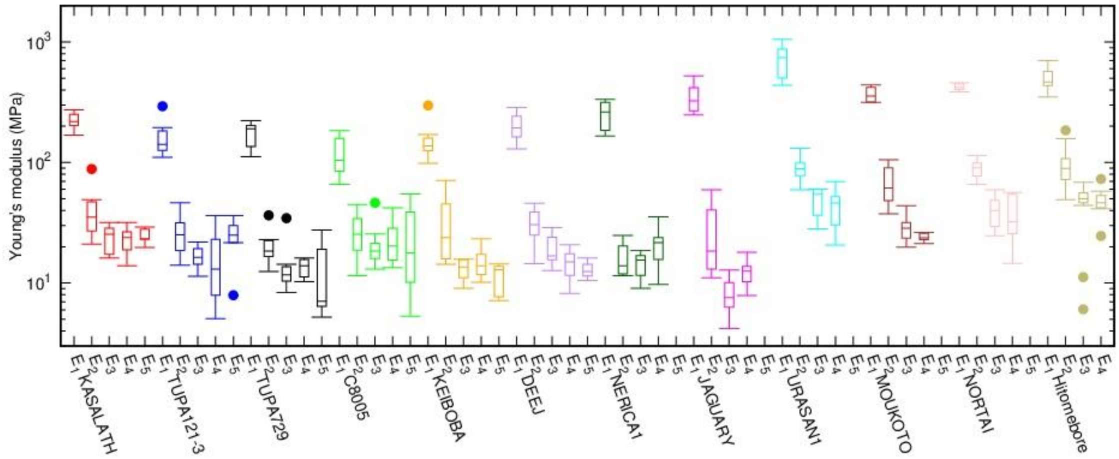

Supplementary Fig. S9. Bending modulus for all the data. D_i denotes the bending modulus of the internode i.

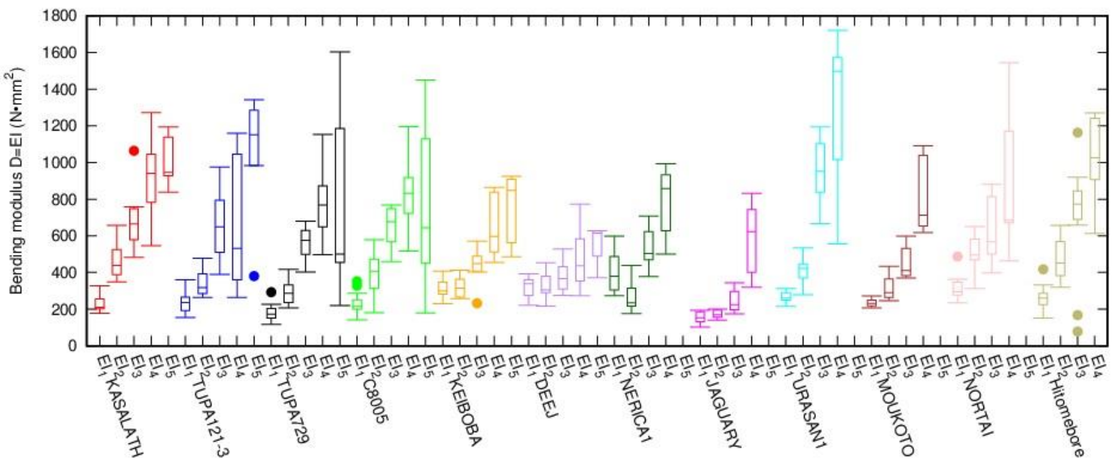

Supplementary Fig. S10. Ear weight for all the data. We_i denotes the ear weight of the internode i.

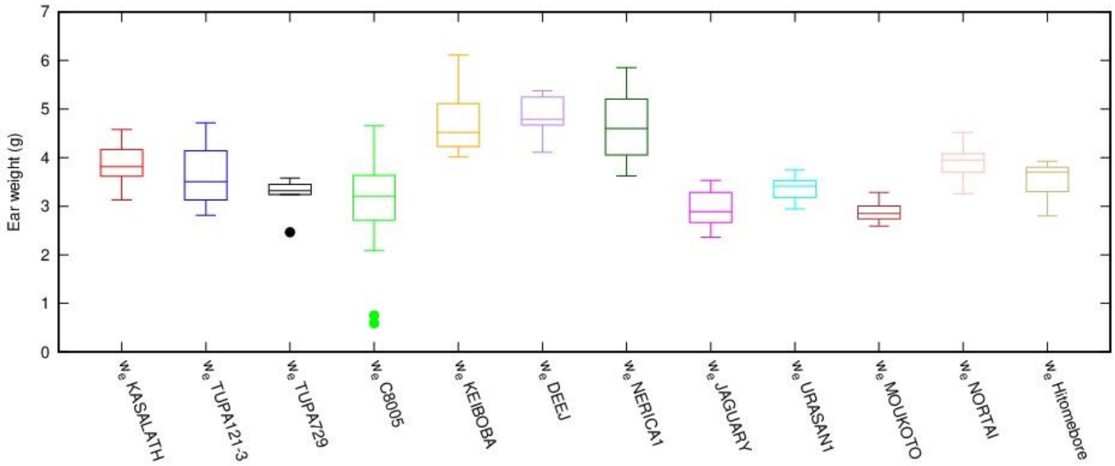

Supplementary Fig. S11. Maximum point loading for all the data. MPL_i denotes the maximum point loading of the internode i.

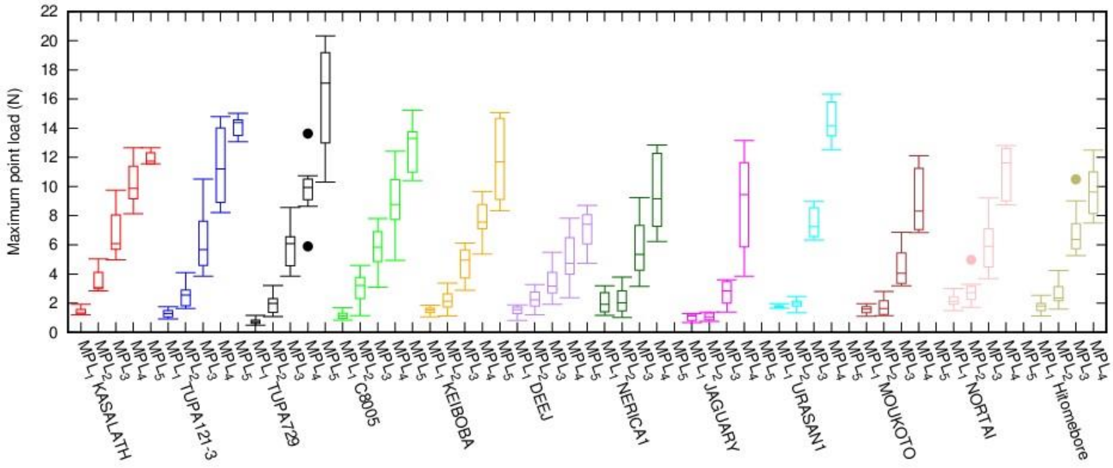

Supplementary Fig. S12. Maximum point displacement for all the data. MPD_i denotes the maximum point displacement of the internode i.

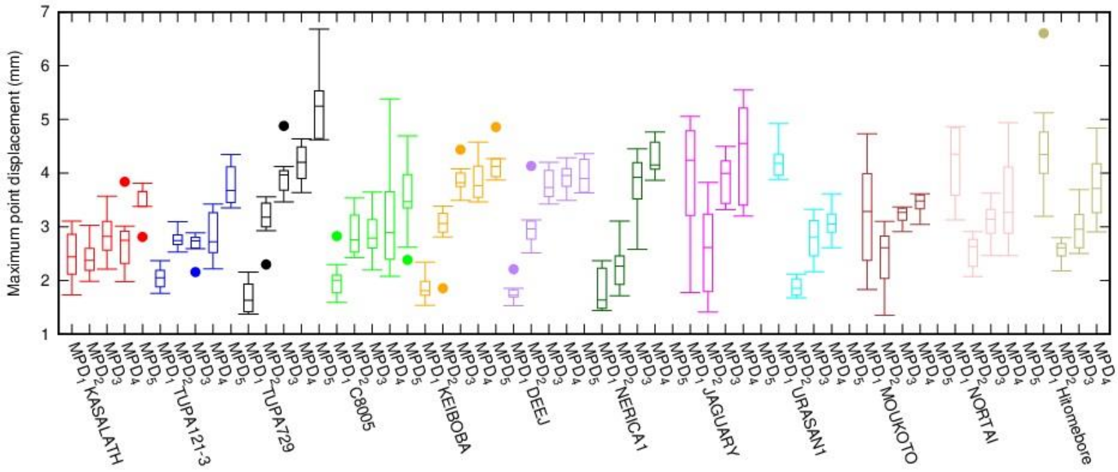

Supplementary Fig. S13. Bending modulus for all the internodes for group A and for group B.

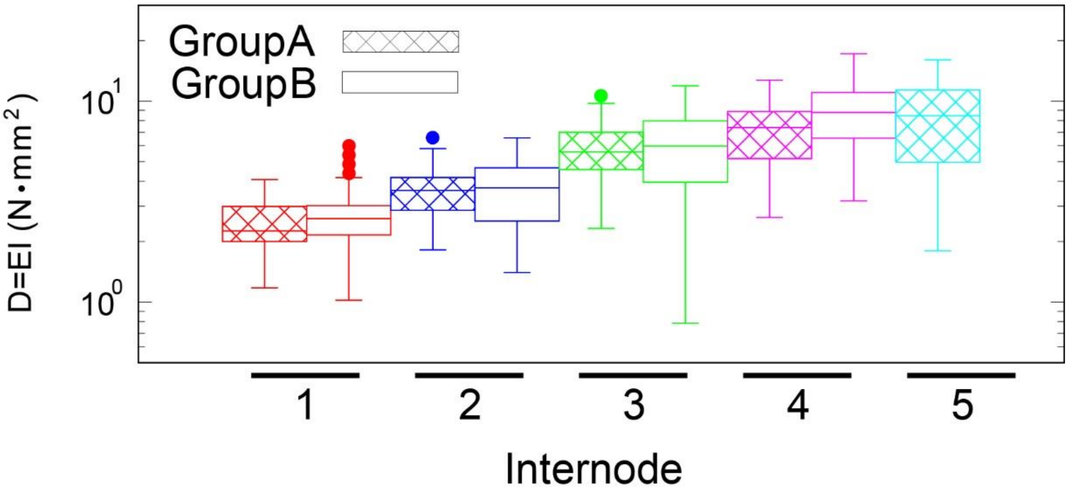

Table S1. List of the accession, origin, category, and WRC No. of rice species. (1) World Rice Core Collection (WRC). (2) New Rice for Africa (NERICA). We referred to the National Institute of Agrobiological Sciences[36].

Accession	Origin	Category	WRC No.[(1)]
KASALATH	India	*aus*	WRC 02
TUPA 121-3	Bangladesh	*aus*	WRC 32
C8005	India	*aus*	
KEIBOBA	China	*indica*	WRC 17
DEEJIAOHUALUO	China	*indica*	WRC 98
NERICA1[(2)]	West Africa	*O. sativa/O. glaberrima*	
JAGUARY	Brazil	Tropical *japonica*	WRC 47
URASAN1	Japan	Tropical *japonica*	WRC 51
TUPA 729	Bangladesh	Tropical *japonica*	WRC 55
MOUKOTO	China	Temperate *japonica*	
NORTAI	U.S.A.	Temperate *japonica*	
Hitomebore	Japan	Temperate *japonica*	

Maclaurin series expansion of the inclination angle: the case of $\frac{d\phi_i}{ds_i} = 0$

We note that $\theta_i(s_i = 0)$ is denoted by ϕ_i in the following formulation.

$$\frac{d\phi_i}{ds_i} = 0,$$

$$\frac{d^2\phi_i}{ds_i^2} = -\alpha_i \sin\phi_i,$$

$$\frac{d^3\phi_i}{ds_i^3} = -\beta_i \sin\phi_i,$$

$$\frac{d^4\phi_i}{ds_i^4} = -\frac{\alpha_i^2}{2}\sin 2\phi_i,$$

$$\frac{d^5\phi_i}{ds_i^5} = 2\alpha_i\beta_i \sin 2\phi_i,$$

$$\frac{d^6\phi_i}{ds_i^6} = 2\beta_i^2 \sin 2\phi_i + \alpha_i^3(2\sin\phi_i - \sin 3\phi_i),$$

$$\frac{d^7\phi_i}{ds_i^7} = \frac{\alpha_i^2\beta_i}{2}(33\sin\phi_i - 17\sin 3\phi_i),$$

$$\frac{d^8\phi_i}{ds_i^8} = \frac{\alpha_i}{2}\sin\phi_i\,(42\beta_i^2 - 15\alpha_i^3\cos\phi_i - 98\beta_i^2\cos 2\phi_i + 17\alpha_i^3\cos 3\phi_i),$$

$$\frac{d^9\phi_i}{ds_i^9} = -\beta_i \sin\phi_i\,(21\beta_i^2 - 108\alpha_i^3\cos\phi_i - 49\beta_i^2\cos 2\phi_i + 124\alpha_i^3\cos 3\phi_i).$$

Maclaurin series expansion of the inclination angle: the case of $\frac{d\phi_i}{ds_i} = c$

$$\frac{d\phi_i}{ds_i} = c,$$

$$\frac{d^2\phi_i}{ds_i^2} = -\alpha_i \sin\phi_i,$$

$$\frac{d^3\phi_i}{ds_i^3} = -\beta_i \sin\phi_i - \alpha_i\phi_i'\cos\phi_i,$$

$$\frac{d^4\phi_i}{ds_i^4} = \alpha_i(\phi_i'^2\sin\phi_i - \phi_i''\cos\phi_i) - 2\beta_i\phi_i'\cos\phi_i,$$

$$\frac{d^5\phi_i}{ds_i^5} = -\alpha_i\left(\phi_i'''\cos\phi_i - \phi_i'^3\cos\phi_i - 3\phi_i'\phi_i''\sin\phi_i\right) - 3\beta_i(\phi_i''\cos\phi_i - \phi_i'^2\sin\phi_i),$$

$$\frac{d^6\phi_i}{ds_i^6} = -\alpha_i\left(\phi_i'''' \cos\phi_i - 3\phi_i''^2 \sin\phi_i - 4\phi_i'''\phi_i' \sin\phi_i - 6\phi_i'^2\phi_i'' \cos\phi_i\right) - 4\beta_i(\phi_i''' \cos\phi_i - \phi_i'^3 \cos\phi_i - 3\phi_i'\phi_i'' \sin\phi_i),$$

$$\frac{d^7\phi_i}{ds_i^7} = -\alpha_i\left(\phi_i''''' \cos\phi_i + \phi_i'^5 \cos\phi_i - 5\phi_i''''\phi_i' \sin\phi_i - 10\phi_i'''\phi_i'' \sin\phi_i - 10\phi_i'''\phi_i'^2 \cos\phi_i + 10\phi_i'^3\phi_i'' \sin\phi_i - 15\phi_i'\phi_i''^2 \cos\phi_i\right) - 5\beta_i(\phi_i'''' \cos\phi_i - 4\phi_i''^2 \sin\phi_i + \phi_i'^4 \sin\phi_i - 4\phi_i'''\phi_i' \sin\phi_i - 6\phi_i'^2\phi_i'' \cos\phi_i),$$

$$\frac{d^8\phi_i}{ds_i^8} = -\alpha_i\Big(\phi_i^{(6)} \cos\phi_i - 10\phi_i'''^2 \sin\phi_i - 15\phi_i''^3 \cos\phi_i - \phi_i'^6 \sin\phi_i - 6\phi_i^{(5)}\phi_i' \sin\phi_i - 15\phi_i^{(4)}\phi_i'' \sin\phi_i - 15\phi_i''''\phi_i'^2 \cos\phi_i + 20\phi_i^{(3)}\phi_i'^3 \sin\phi_i + 45\phi_i'^2\phi_i''^2 \sin\phi_i + 15\phi_i'^4\phi_i'' \cos\phi_i - 60\phi_i^{(3)}\phi_i'\phi_i'' \cos\phi_i\Big) - 6\beta_i(\phi_i^{(5)} \cos\phi_i + \phi_i'^5 \cos\phi_i - 5\phi_i^{(4)}\phi_i' \sin\phi_i - 10\phi_i'''\phi_i'' \sin\phi_i - 10\phi_i'''\phi_i'^2 \cos\phi_i + 10\phi_i'^3\phi_i'' \sin\phi_i - 15\phi_i'\phi_i''^2 \cos\phi_i),$$

$$\frac{d^9\phi_i}{ds_i^9} = -\alpha_i\Big(\phi_i^{(7)} \cos\phi_i - \phi_i'^7 \cos\phi_i - 7\phi_i^{(6)}\phi_i' \sin\phi_i - 21\phi_i^{(5)}\phi_i'' \sin\phi_i - 21\phi_i^{(5)}\phi_i'^2 \cos\phi_i - 35\phi_i'''\phi_i^{(4)} \sin\phi_i + 35\phi_i^{(4)}\phi_i'^3 \sin\phi_i - 105\phi_i^{(3)}\phi_i''^2 \cos\phi_i + 35\phi_i^{(3)}\phi_i'^4 \cos\phi_i - 70{\phi_i^{(3)}}^2\phi_i' \cos\phi_i - 21\phi_i^{(5)}\phi_i'' \sin\phi_i + 105\phi_i'\phi_i''^3 \sin\phi_i + 105\phi_i'^3\phi_i''^2 \cos\phi_i - 105\phi_i^{(4)}\phi_i'\phi_i'' \cos\phi_i + 210\phi_i^{(3)}\phi_i'^2\phi_i''\Big) - 7\beta_i(\phi_i^{(6)} \cos\phi_i - 10{\phi_i^{(3)}}^2 \sin\phi_i - 15\phi_i''^3 \cos\phi_i - \phi_i'^6 \sin\phi_i - 6\phi_i^{(5)}\phi_i' \sin\phi_i - 15\phi_i^{(4)}\phi_i'' \sin\phi_i - 15\phi_i^{(4)}\phi_i'^2 \cos\phi_i + 20\phi_i^{(3)}\phi_i'^3 \sin\phi_i + 45\phi_i'^2\phi_i''^2 \sin\phi_i + 15\phi_i'^4\phi_i'' \cos\phi_i - 60\phi_i^{(3)}\phi_i'\phi_i'' \cos\phi_i)$$
